# Carvacrol derivatives as mushroom tyrosinase inhibitors; synthesis, kinetics mechanism and molecular docking studies

**DOI:** 10.1371/journal.pone.0178069

**Published:** 2017-05-23

**Authors:** Zaman Ashraf, Muhammad Rafiq, Humaira Nadeem, Mubashir Hassan, Samina Afzal, Muhammad Waseem, Khurram Afzal, Jalifah Latip

**Affiliations:** 1 Department of Chemistry, Allama Iqbal Open University, Islamabad, Pakistan; 2 Department of Biochemistry & Biotechnology (Baghdad-ul-Jadeed Campus), The Islamia University of Bahawalpur, Bahawalpur, Pakistan; 3 Deapertment of Pharmaceutical Chemistry, Riphah Institute of Pharmaceutical Sciences, Riphah International University, Islamabad, Pakistan; 4 Department of Biology, College of Natural Sciences, Kongju National University, Gongju, Republic of Korea; 5 Department of Pharmacy, Faculty of Pharmacy, BahauddinZakria University, Multan Pakistan; 6 Department of Biology, Allama Iqbal Open University, Islamabad, Pakistan; 7 School of Chemical Sciences & Food Technology, Faculty of Science and Technology, Universiti Kebangsaan Malaysia, Bangi, Selangor, Malaysia; Aligarh Muslim University, INDIA

## Abstract

The present work describesthe development of highly potent mushroom tyrosinase inhibitor better than the standard kojic acid. Carvacrol derivatives **4a-f** and **6a-d** having substituted benzoic acid and cinnamic acidresidues were synthesized with the aim to possess potent tyrosinase inhibitory activity.The structures of the synthesized compounds were ascertained by their spectroscopic data (FTIR, ^1^HNMR, ^13^CNMR and Mass Spectroscopy).Mushroom tyrosinase inhibitory activity of synthesized compounds was determined and it was found that one of the derivative **6c** possess higher activity (IC_50_ 0.0167μM) than standard kojic acid (IC_50_ 16.69μM). The derivatives **4c** and **6b** also showed good tyrosinase inhibitory activity with (IC_50_ 16.69μM) and (IC_50_ 16.69μM) respectively.Lineweaver—Burk and Dixon plots were used for the determination of kinetic mechanism of the compounds **4c** and **6b** and **6c**. The kinetic analysis revealed that compounds **4c** and **6b** showed mixed-type inhibition while **6c** is a non-competitive inhibitor having *Ki* values19 μM, 10 μM, and 0.05 μMrespectively. The enzyme inhibitory kinetics further showed thatcompounds **6b** and **6c** formed irreversible enzyme inhibitor complex while **4c** bind reversibly with mushroom tyrosinase.The docking studies showed that compound **6c** have maximum binding affinity against mushroom tyrosinase (PDBID: 2Y9X) with binding energy value (-7.90 kcal/mol) as compared to others.The 2-hydroxy group in compound **6c** interacts with amino acid HIS85 which is present in active binding site. The wet lab results are in good agreement with the dry lab findings.Based upon our investigation we may propose that the compound **6c** is promising candidate for the development of safe cosmetic agent.

## Introduction

Tyrosinase a copper containing metalloenzymemediating the o-hydroxylation of monophenols to catechols and the subsequent two-electron oxidation to quinines [1]. The physiological function of tyrosinase is to convert tyrosine into dopaquinone, which represents the first step of melaninbiosynthesis in melanosomes [2–3].The key starting material for melanin biosynthesis is aromatic amino acid L-tyrosine [4].The L-tyrosine and L-3,4-dihydroxyphenylalanin (L-DOPA) play vital role in regulation of the melanin synthesis [5–7]. The color of human skin is determined by the presence of melanin in the surrounding keratinocytes [8–9]. A number of other factors such as UV exposure, α-melanocyte-stimulating hormone, melanocortin 1 receptor and agouti-related protein are also involved in melanogenesis [10–11].The melanogenesiscorrespondsto a possible cellular danger and is confined to special melanosomes in melanocytes, which synthesize pigments and transfer them to recipient cells [12].The abnormal proliferation of melanocytes produced melanoma a type of skin cancer[13–14].

The abnormal accumulation of melaninin keratinocytes caused melasma and post-inflammatory disorders for which patients pursue treatment [15].A number of other melanocytedisorders like senile lentigo, freckles and pigmented acne scars occur in human of all races worldwide[16].Hyperpigmetationdisorders adversely affect person’s psychological and social well-beingwhich results in lower output, overall performance, and self-confidence [17].Tyrosinasecan also be linked to neurodegenerative diseases owing to excessive production ofdopaquinonesbyoxidation of dopamine results in neuronal damage and cell death [18–20].It has also been reported that tyrosinase is responsible for quicker degradation and lesser shelf life of fruits and vegetables during postharvest handling and processing [21–23].The therapeutics used currently to treat hyperpigmentation disorders generally associated with side effects. Thus synthesis of potent tyrosinase inhibitors with minimal side effects is of great interest in the medical, agricultural and cosmetic industries.

The antioxidant and tyrosinase inhibitory activities of substituted benzoic and cinnamic acids have been reported previously [24–25]. Carvacrol is a naturally occurring monoterpene phenol present in thyme along with thymol. Carvacrol besides its odoriferous functions exhibited antimicrobial activities [26–28]. Thus, carvacrol and thymol are employed as meat preservatives or flavoring agents in the food industry. The antioxidant activity of thyme essential oils was previously reported, and it has been identified to be due to the presence of carvacrol and thymol [29–31]. Carvacrol as antioxidant protects food qualities and organisms from damage induced by oxidative stress. In contrast to these well-studied biological effects the tyrosinase inhibitory potential of carvacrol is poorly understood. The present work describes the synthesis, tyrosinase inhibitory kinetics and computation studies of carvacrol derivatives. The title compounds were synthesized by incorporating the substituted benzoic and cinnamic acids. The enzyme inhibitory kinetics of the most potent derivatives was also determined. Molecular docking studies were also carried to compare the wet lab results with the computational results.

## Results and discussion

### Chemistry

The carvacrol derivatives **4a-f** and **6a-d** were obtained by following the previouslydescribed method [32] with slight modification shown insupporting informations (S1 and S2 Figs). The carvacrol in the first step is converted in tointermediate **2** by esterification reaction with chloroacetyl chloride in the presence of (C_2_H_5_)_3_N and anhydrous methylene chloride as solvent. The formation of the intermediate **2** was ascertained by the presence of ester carbonyl stretching at 1731cm^-1^ and disappearance of the—OH stretching in FTIR spectra. The title carvacrol derivatives **4a-f** and **6a-d** were synthesized by simple nucleophilic replacement of chloro group from intermediate **(2)** by carboxylic group (-COOH) of substituted benzoic acids **3a-f** and cinnamic acids **5a-d** respectively. The structures of the final products were confirmed by FTIR, ^1^HNMR, ^13^CNMR and Mass spectroscopic data.

### Bioassay for tyrosinase inhibitory activity

The substituted thymol and vanillin based tyrosinase inhibitors have already been reported by the author and got comparable IC_50_ values with the standard kojic acid repeated. We have evaluated the role of alkyl, methoxy and aldehyde functional groups in tyrosinase inhibitory activity. In this paper carvacrol derivatives **4a-f** and **6a-d** were designed based upon our previous investigations. This time 2,4-dihydroxy cinnamic acid moiety is also attached with the carvacrol and we got excellent activity compared to standard kojic acid. The hydroxy substituted benzoic acids and cinnamic acids were selected to synthesize derivatives **4a-f** and **6a-d** respectively. Thehydroxy substituted tyrosinase inhibitors with IC_50_ value less than 10μM are of special interest. The well known clinically used tyrosinase inhibitor kojic acid was used as reference drug for comparison purpose. The bioassay results showed that hydroxy substituted cinnamic acid moiety play major role in tyrosinase inhibitory activity. The substitution pattern of hydroxyl groups at phenyl ring is the decisive factor of inhibitory activity. Thecompound **6c** bearing 2,4-dihydroxy substituted cinnamic acid residue exhibited excellent tyrosinase inhibitory activity (IC_50_ 0.0167μM).The compounds which possess hydroxy substituted cinnamic acid residue are in general are more active than those having hydroxy substituted benzoic acid moiety (Table 1). The derivatives **4a** and **4b** having mono hydroxy substituted benzoic acid ring showed comparable activity (IC_50_ 14.9μM) to that ofkojic acid (IC_50_ 16.69μM).On the other hand the compound **4c** having 2,4-dihydroxy substituted benzoic acid residue exhibited higher tyrosinase inhibitory activity (IC_50_ 6.7μM) than kojic acid. The compounds **6b** and **6d** possessing substituted cinnamic acid functionalities showed IC_50_ values 6.5 and 6.7 μM respectively better than kojic acid. Interestingly, carvacrol derivative **6c** bearing 2,4-dihydroxy substituted cinnamic acid moiety showed most potent activity than all of the synthesized compounds and kojicacid.We may propose that the hydroxy substitution pattern on phenyl ring in case of compound **6c** impedes the molecule to interact well with the active sites of enzyme. The kinetic analysis and docking studies helps us to explore the mode of binding and residual interactions between enzyme and synthesized inhibitors.

**Table 1:** Tyrosinase inhibitory activity of carvacrol derivatives (4a-f) and (6a-d).

Compounds	Mushroom Tyrosinase Inhibition IC_50_ (μM)
**4a**	14.9 ± 0.91
**4b**	14.9 ± 2.13
**4c**	6.7 ± 1.20
**4d**	15.9 ± 3.71
**4e**	93.8 ± 9.32
**4f**	65.2 ± 3.89
**6a**	7.7 ± 0.70
**6b**	6.5 ± 0.35
**6c**	0.0167 ± 0.0011
**6d**	6.7 ± 1.34
**Kojic acid**	16.69 ± 2.8

#### Kinetic studies

**Fig 1:**
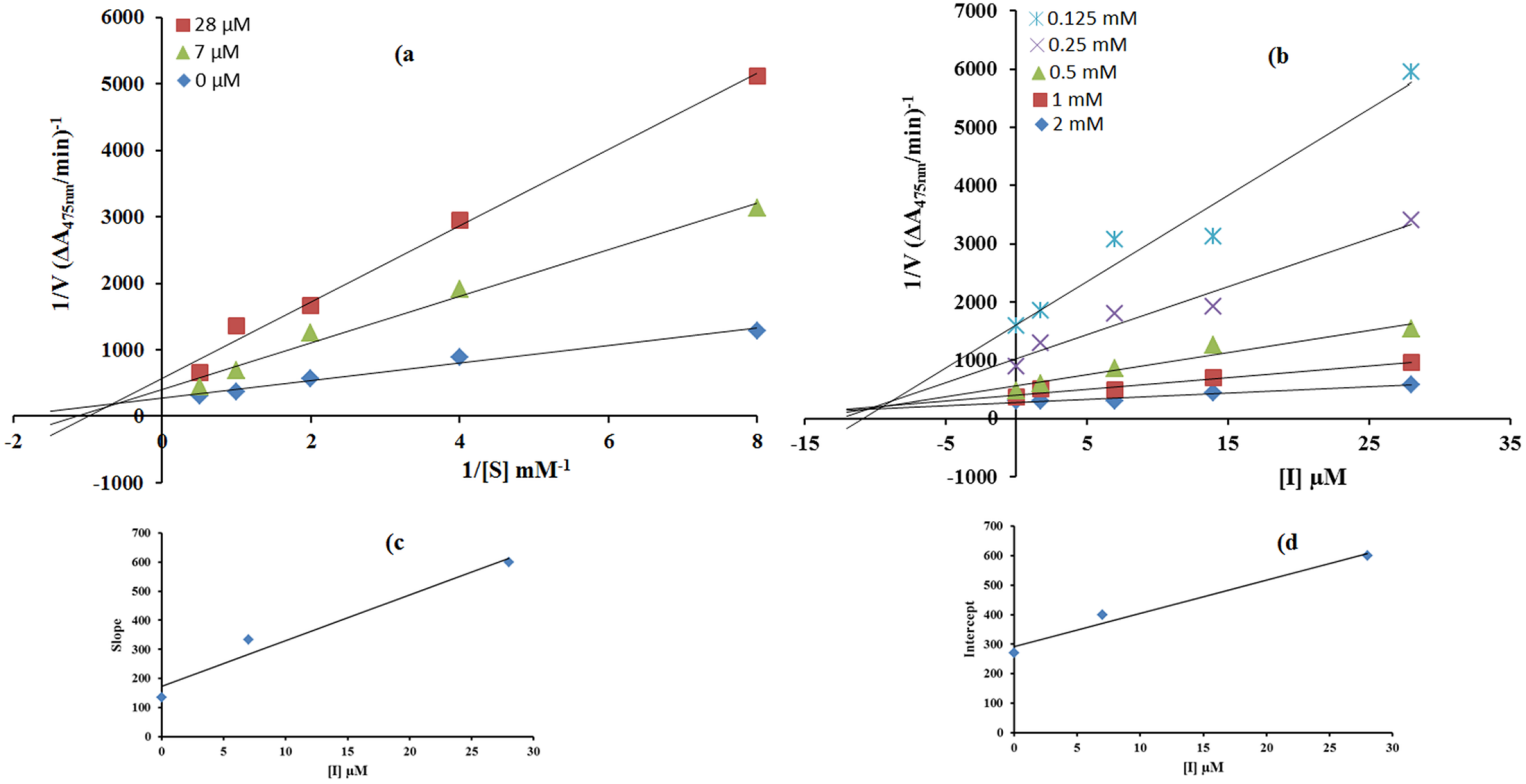
**a)** Determination of inhibition type of compound **(6b)** by Lineweaver-Burk plots **c,d)** the insets represent the plot of the slope & intercept respectively, from Lineweaver-Burk plot versus inhibitor of **(6b)** with concentrations (0.0, 7.0 and 28 μM). **b)** Determination of inhibition constant of compound **(6b)** with concentrations (0.0, 1.75, 7.0, 14.0 and 28 μM) by Dixon plots.The concentration of L-DOPA was 0.125 to 2.00 mM in both cases.

**Fig 2:**
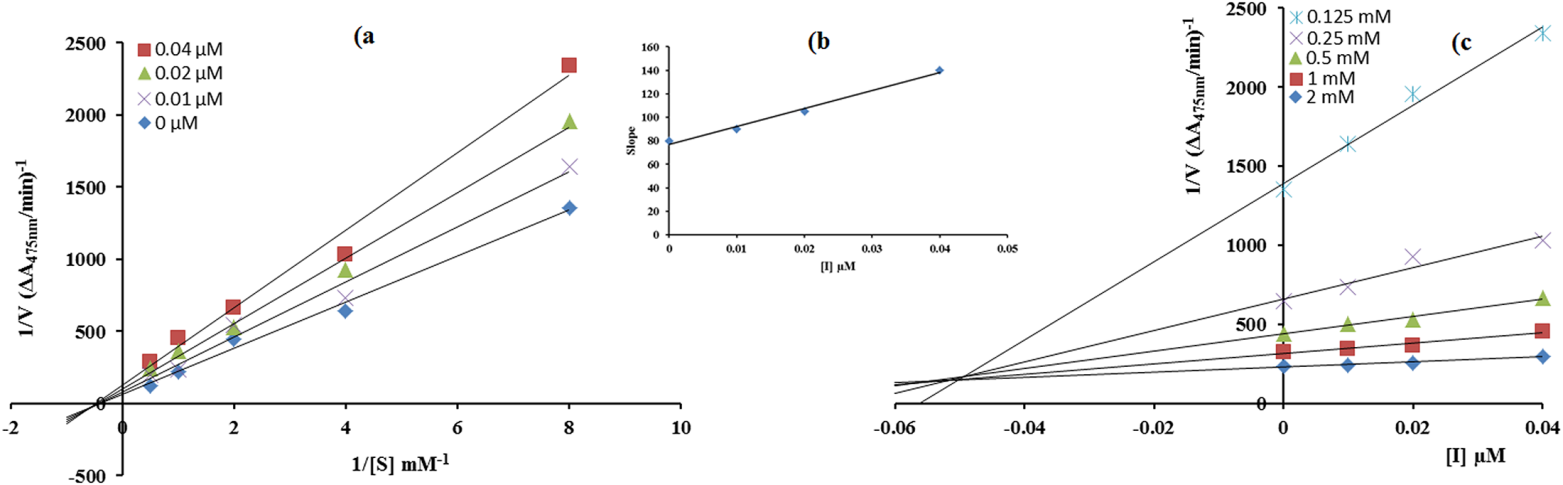
a) Determination of inhibition type of compound (6c) by Lineweaver-Burk plots b) the insets represents the plot of the slope from Lineweaver-Burk plot versus inhibitor of (6c)c) Determination of inhibition constant of compound (6c) by Dixon plots. The concentrations of compound **(6c)** (0.0, 0.01, 0.02 and 0.04μM) and the concentrations of L-DOPA were (0.125, 0.25, 0.5, 1.0 and 2.00 mM) used in both cases.

**Fig 3:**
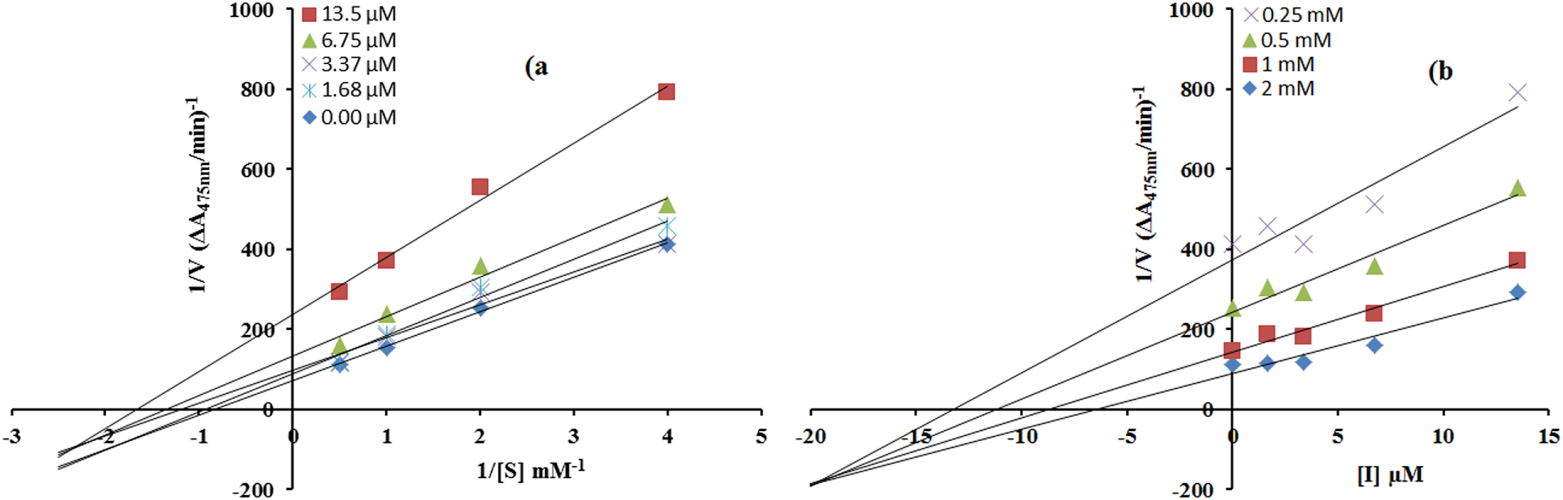
a) Determination of inhibition type of compound (4c)by Lineweaver-Burk plots b) Determination of inhibition constant of compound (4c) by Dixon plots. The concentrations of compound **(4c)** (0.0, 1.68, 3.37, 6.75 and 13.5μM) and the concentrations of L-DOPA were (0.25, 0.5, 1.0 and 2.00 mM) used in both cases.

The mode of inhibition of synthesized carvacrol derivatives **4c, 6b** and **6c** on the diphenolase activity, during the oxidation of L-DOPA, was determined from Lineweaver—Burk and Dixon plots. In the presence of compounds **6b**, **6c** and **4c** the kinetics of the enzyme was shown in Figs 1(a)–3(a). The plots of *1/V* versus 1/ [S] gave a family of straight lines with different slopes. The analysis showed that *V*_max_ decreased with changing *K*_*m*_ in the presence of increasing concentrations of compound **6b** and **4c** which indicated that both of thesecompoundsinhibit tyrosinase by two different pathways, and show mixed type inhibition. This result showed that compounds **6b** and **4c** can bind, not only with free enzyme, but also with the enzyme-substrate complex [33]. The kinetic study of most active compound **6c** showed that it is a non-competitive inhibitor of mushroom tyrosinase Fig 2(a). The value of 1/*V*_max_is increased to a new value while that of *K*_m_ remains same which indicated that the compound **6c** simply lowers the concentration of functional enzyme by non-competitively binding mode at enzyme [34]. On the other hand in Dixon plot, slope obtained from the plots for uninhibited enzyme and with different concentrations of inhibitors **6b**, **6c** and **4c** was consistent with the characteristic patterns of inhibition of compounds **6b**, **6c** and **4c** with *Ki* value 10 μM, 0.05 μM and 19 μM as shown in Figs 1(b)–3(b), respectively.The inhibition constant *Ki* for compound **6c** was also calculated by secondary replot of slope from Lineweaver-Bruk plot versus inhibitor concentrations as shown in Fig 2(b).

The behavior of compound **6b** indicated that it inhibits tyrosinase by two different pathways; competitively forming enzyme-inhibitor (EI) complex and interrupting enzyme-substrate-inhibitor (ESI) complex in non-competitive manner. Binding affinities of EI and ESI complexes were determined to gain insightful pathway in which compounds **6b** preferentially preceded, the secondary replots of slope versus concentration of compounds **6b** showed EI dissociation constant Fig 1(c), while ESI dissociation constant was shown by secondary replots of intercept versus concentration of compounds **6b** Fig 1(d). A lower dissociation constant of *Ki*pointed out stronger binding between enzyme and compounds **6b** which suggested preferred competitive over noncompetitive manners [35].

#### The inhibitory effect of compounds (6b), (6c) and (4c) on diphenolase activity of tyrosinase

**Fig 4:**
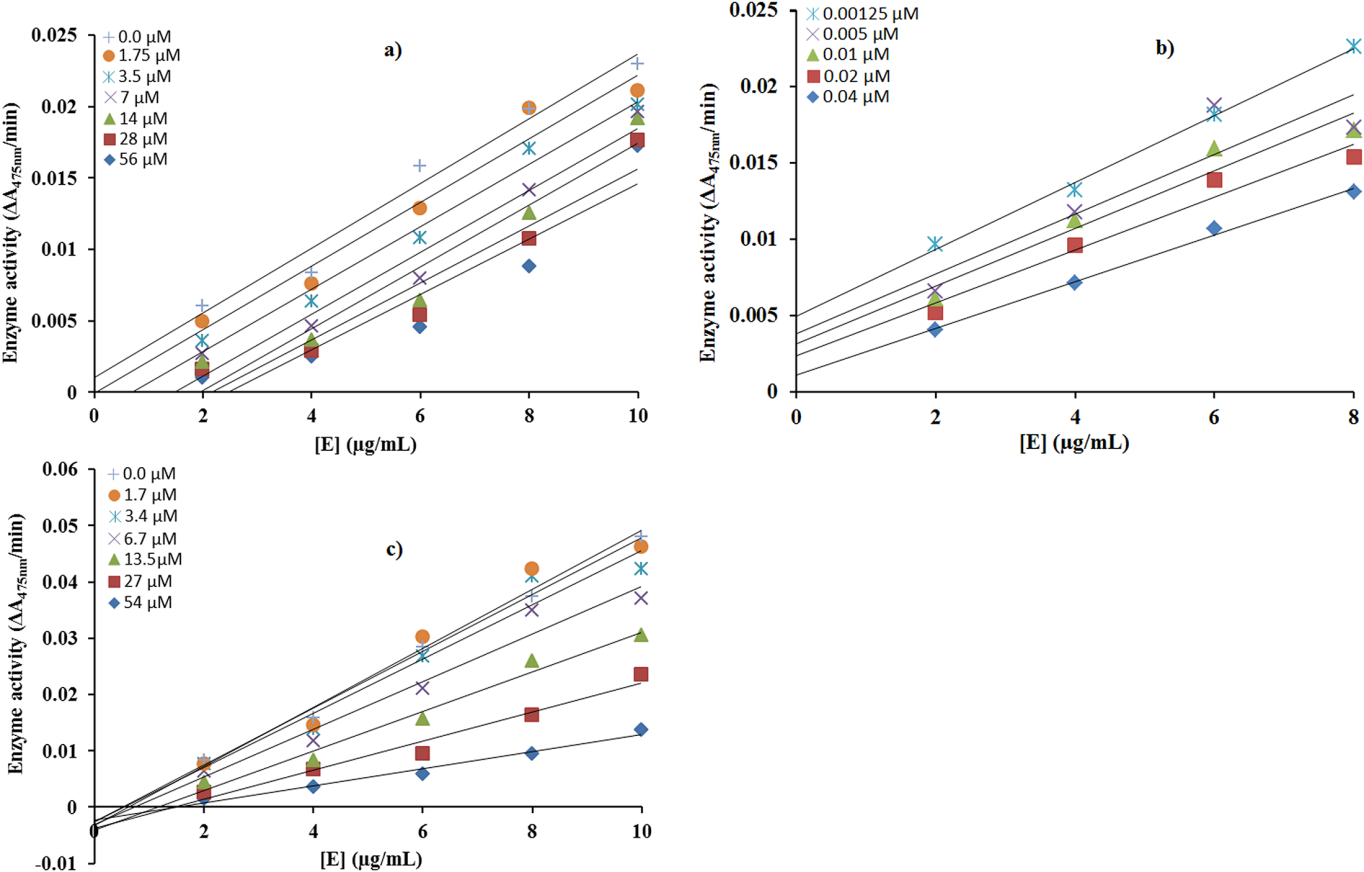
a,b,c) Relationship between the catalytic activity of tyrosinase and various concentrations of compounds (6b), (6c) and (4c) respectively.

The inhibitory mechanism of mushroom tyrosinase by compounds **6b**, **6c** and **4c** for the oxidation of L-DOPAwas studied as shown in Fig 4(a), 4(b) and 4(c), respectively. The plots of the remaining enzyme activity versus the concentration of enzyme (2, 4, 6, 8 and 10 μg/mL) in the presence of different concentrations of compound **6b**, **6c** and **4c** for the catalysis of L-DOPA gave a series of straight lines.In this study, we found the parallel straight lines with the same slopes, indicating that the inhibitory effect of compounds **6b** and **6c** was irreversible [36–37]to the enzyme Fig 4(a) and 4(b). These results suggested that both the compounds **6b** and **6c** effectively inhibited the enzyme by binding to its binuclear active site irreversibly. In case of compound **4c** all the lines intersected at the same point on X-axis. Increasing the inhibitor concentration resulted in a decrease in the slope of the lines, which indicated that the compound **4c** was a reversible inhibitor of tyrosinase [38](Fig 4(c).

### Structural assessment of mushroom tyrosinase

Mushroom tyrosinase (Agaricusbisporus) is a class of oxidoreductase copper containing protein comprises 391 amino acids. The structure architecture of mushroom tyrosinase showed that, it consists of 39% helial (154 residues) and 14% β sheet (57 residues) and 46% coil (180 residues). The X-Ray diffraction study confirmed its resolution 2.78Å, R-value 0.238 and unit cell length values were observed for a = 103.84, b = 104.82 and c = 119.36 with angles 90°, 110.45° and 90° for all α, β and γ dimensions respectively. The Ramachandran plots and values indicated that 95.90% of residues were in favored regions and 100.0% residues were lies in allowed regions. This selected Ramachandran graph values showed the good accuracy of phi (φ) and psi (ψ) angles among the coordinates of receptor molecules and most of residues plummeted in acceptable region.

### Molecular docking analysis

The molecular docking studies of the synthesized compounds **4a-f** and **6a-d** were performed against mushroom tyrosinase (PDBID: 2Y9X) to determine their binding affinities. The 2,4-dihydroxy substituted derivatives showed best affinity for the target protein compared to monosubstituted, 3,4- and 3,5- dihydroxy substituted ones. The docking results displayed that derivatives having hydroxy substituted cinnamic acids functionality exhibited highest affinity than all other synthesized compounds Table 2.

**Table 2:** Docking results of carvacrol derivatives (4a-4f) and (6a-6d) using AutoDock.

Comps	Binding energy (kcal/mol)	Ligand efficiency	Inhibition constant (uM)	Intermolecular energy (kcal/mol)	Vdw_hb_desolve_energy (kcal/mol)	Electrostatic energy (kcal/mol)	Torsional energy (kcal/mol)
**4a**	-7.40	-0.27	14.78	-9.27	-9.16	-0.12	2.68
**4b**	-7.50	-0.27	15.78	-9.24	-9.05	-0.19	2.68
**4c**	-7.80	-0.24	36.03	-9.04	-8.80	-0.25	2.98
**4d**	-7.30	-0.25	21.38	-9.35	-9.09	-0.26	2.98
**4e**	-7.20	-0.26	18.42	-9.44	-9.32	-0.13	2.98
**4f**	-7.70	-0.24	32.46	-9.40	-9.11	-0.29	3.28
**6a**	-7.70	-0.28	7.50	-9.97	-9.86	-0.11	2.98
**6b**	-7.80	-0.25	14.43	-9.89	-9.89	-0.05	3.28
**6c**	-7.90	-0.21	63.99	-9.30	-9.23	-0.07	3.58
**6d**	-7.50	-0.24	24.41	-9.28	-9.10	-0.18	2.98

**Fig 5:**
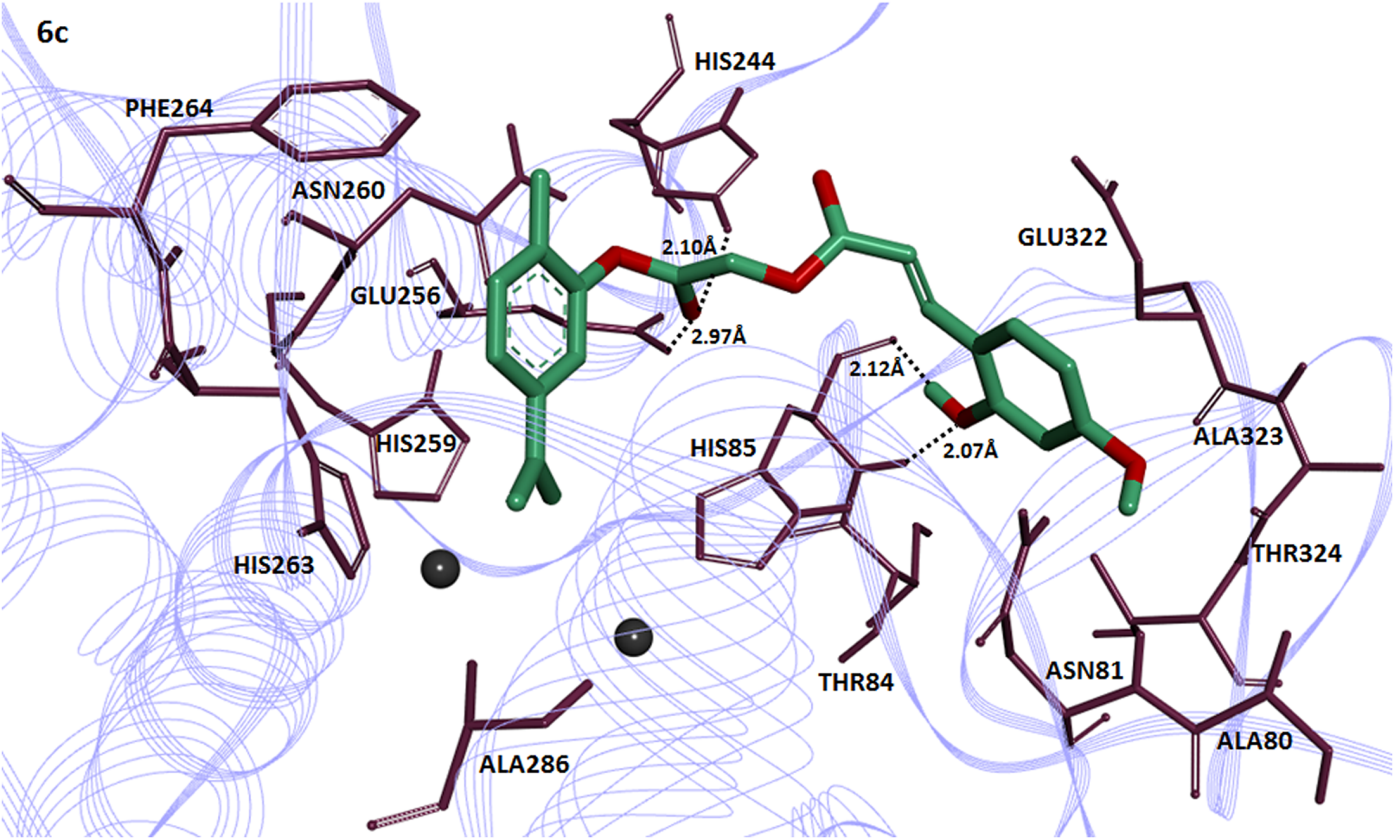
Binding interactions of compound (6c) with target protein. The **(6c)** is mentioned in green color with embedded oxygen functional groups in red. The target protein is highlighted in line ribbon format with purple color. The active binding site amino acids are highlighted in dark mahroon color around the ligand molecule. Two copper ions are also present in black color.

**Fig 6:**
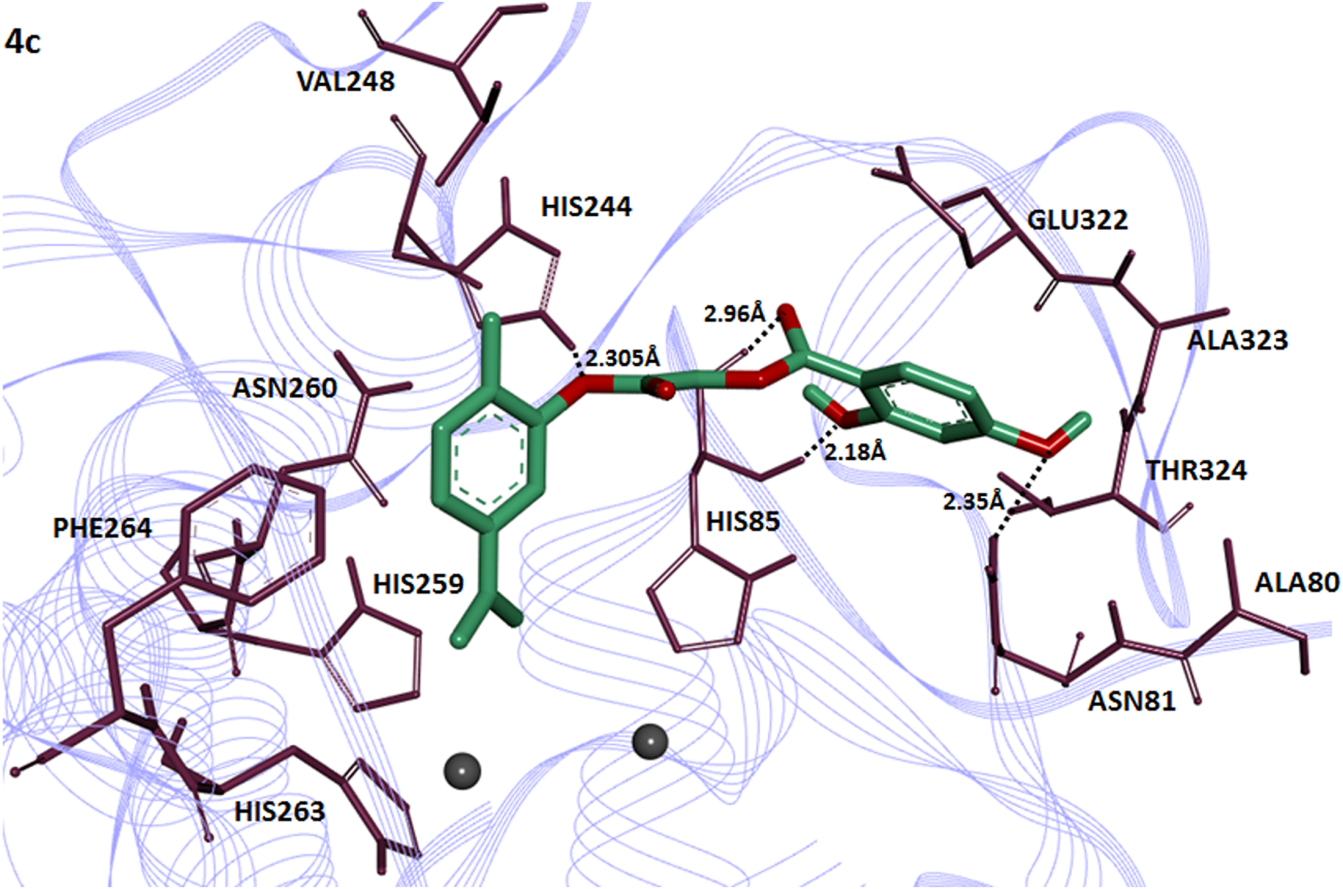
Binding interactions of compound (4c) with target protein. The **(4c)** is mentioned in green color with embedded oxygen functional groups in red. The target protein is highlighted in line ribbon format with purple color. The active binding site amino acids are highlighted in dark mahroon color around the ligand molecule. Two copper ions are also present in black color.

**Fig 7:**
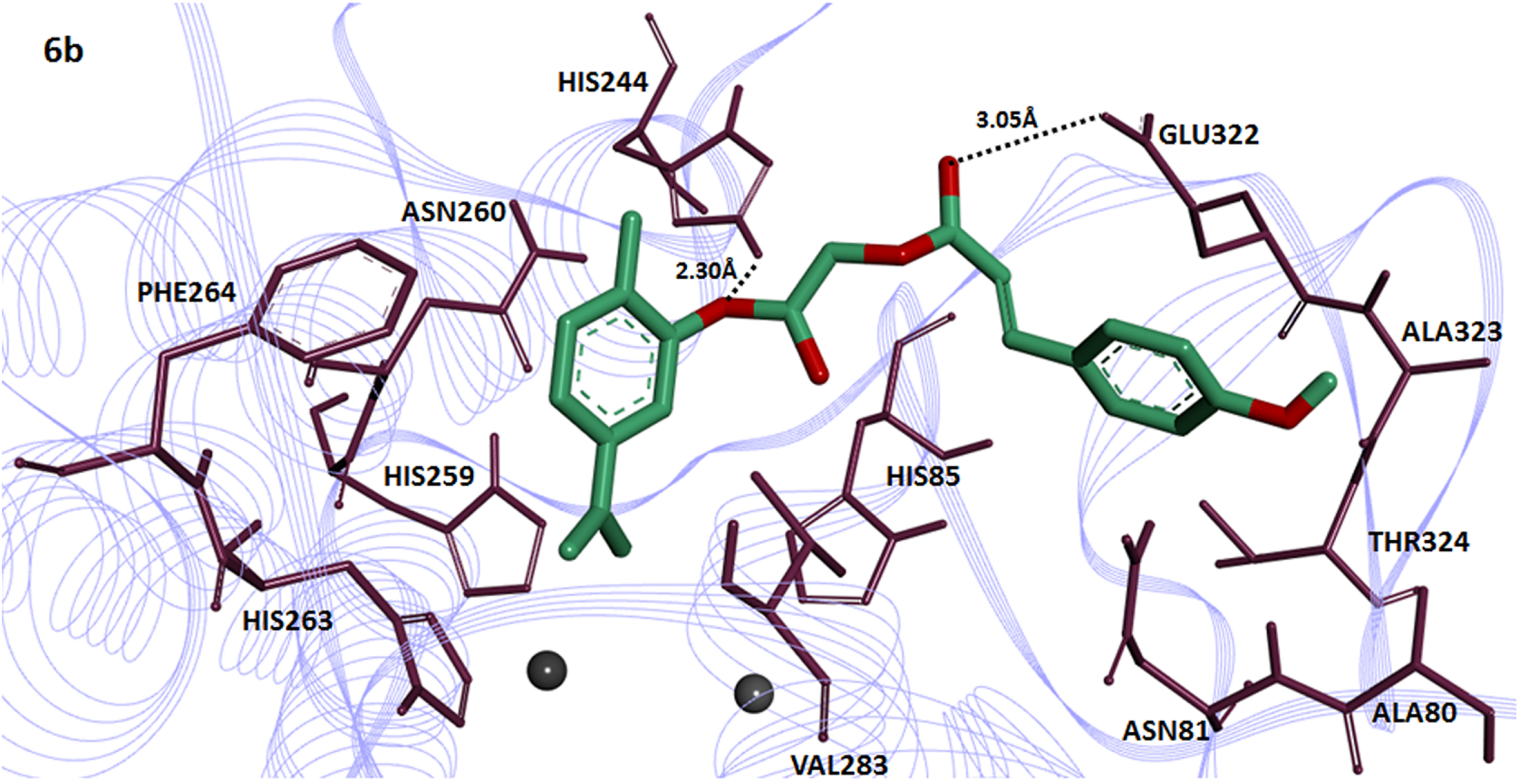
Docking interactions between 6b and target protein. The **6b** is mentioned in green color with embedded oxygen functional groups in red. The target protein is highlighted in line ribbon format with purple color. The active binding site amino acids are highlighted in dark mahroon color around the ligand molecule. Two copper ions are also present in black color.

The compound **6c** having 2,4-dihydroxy substitution showed maximum binding affinity with binding energy value (-7.90 kcal/mol) as compared to others.The 2-hydroxy group of cinnamic acid in **6c** interacts with amino acid HIS85 of the target protein forming two hydrogen bonds with bonding distance 2.07 and 2.12Å. The ester carbonyl oxygen in the same compound also interacts with HIS284 and GLU256 at a bonding distance 2.10 and 2.97Å respectively (Fig 5). It has been reported that these amino acids are located in active binding site and are significant in the downstream signaling pathways [39].The compound **4c** having 2,4-dihydroxy substituted benzoic acid residue showed good binding affinity with binding energy value(-7.80 kcal/mol). Interestingly 2-hydroxy group of benzoic acid moiety in compound **4c** interacts withHIS85 and 4-hydroxy interacts with ASN81having bond length 2.18 and 2.35Å respectively (Fig 6). The Fig 7 depicted the binding interactions of the compound **6b** with the amino acid residue of the target protein. It also possesses good binding affinity (-7.80 kcal/mol). The ester oxygen of the carvacrol ring in compound **6b** interacts with the HIS244 having binding distance 2.30Å.

## Conclusion

The carvacrol derivatives **4a-4f** and **6a-6d** were synthesized by incorporation of the substituted benzoic acids and cinnamic acids to discover the highly potent mushroom tyrosinase inhibitor.The hydroxy substituted derivatives showed good to excellent tyrosinase inhibitory activity. The compound **6c** bearing 2,4-dihydroxy substituted cinnamic acid residue exhibited excellent tyrosinase inhibitory activity (IC_50_ 0.0167μM).The derivatives with hydroxy substituted cinnamic acid residue possess greater tyrosinase inhibitory potential as compared to benzoic acids.The kinetic study of most active compound **6c** showed that it is a non-competitive inhibitor with *Ki* value 0.05 μM.The kinetic results further revealed that derivative **6c** effectively inhibited the enzyme by binding to its binuclear active site irreversibly.The docking studies showed that compound **6c** have maximum binding affinity against mushroom tyrosinase (PDBID: 2Y9X) with binding energy value (-7.90 kcal/mol) as compared to others.The 2-hydroxy group in compound **6c** interacts with amino acid HIS85 which is present in active binding site. Based upon our investigation it is proposed that carvacrol derivative **6c** may have potential to design new cosmetic agents.

## Experimental

All chemicals used for the synthesis of compounds were purchased from Sigma Chemical Co. Melting points were determined using a Digimelt MPA 160, USA melting point apparatus and are reported uncorrected. The FTIR spectra were recorded with Shimadzu FTIR–8400S spectrometer (Kyoto, Japan, υ, cm^-1^). Mass spectra were performed on an Agilent 6460 Series Triple Quadrupole instrument (Agilent). Electrospray ionization both in the positive (ESI+) and negative ion mode (ESI-) was employed for ionization. Elemental Analysis (C, H) were carried out on a Flash 2000 series elemental analyzer with TCD detector system and results are with ±0.3%. The ^1^H NMR and ^13^C NMR spectra (DMSO-*d*_6_) were recorded using a Bruker 400 MHz spectrometer. Chemical shifts (δ) are reported in ppm downfield from the internal standard tetramethylsilane (TMS). The purity of the compounds was checked by thin layer chromatography (TLC) on silica gel plate using n-hexane and ethyl acetate as mobile phase.

### Reagents

Mushroom tyrosinase was purchased from Sigma (USA); L-DOPA and thymol were purchased from Sigma (USA). Stock solutions of the reducing substrates were prepared in phosphate buffer (20 mM, pH 6.8).

### Synthesis of intermediate (2)

Carvacrol **1** (0.01 mol), triethylamine (0.01 mol) were mixed in dry dichloromethane (25mL) at 0 to -5°C. The reaction mixture was treated withchloroacetyl chloride (0.01mol) in dry dichloromethane drop wise with constant stirring over a period of 1h maintaining the temperature constant. The reaction mixture was then stirred at room temperature for further 5hand progress of the reaction was determined by thin layer chromatography. After the completion of reaction the mixture was washed with 5% HCl, and 5% sodium hydroxide solution. The organic layer was washed with saturated aqueous NaCl, dried over anhydrous magnesium sulphate, filtered and solvent was removed under reduced pressure. The crude intermediate **2** was purified by silica gel column chromatography using n-hexane:ethyl acetate (3:1) as eluents. Oil; reaction time, 4h; yield, 86%; R_f_ 0.62 (n-hexane: ethyl acetate 3:1), FTIR ν_max_ cm^-1^: 2965 (sp2 C-H), 2877(sp3 C-H), 1731 (C = O ester), 1587 (C = C aromatic), 1156 (C-O, ester).

#### Synthesis of carvacrol derivatives(4a-f) and (6a-d)

The hydroxy substituted benzoic acids **3a-f** (0.01mole), triethyl amine (0.01 mol), potassium iodide (0.01 mol) in dimethyl formamide (25 mL) and intermediate **2** (0.01 mol)were stirred overnight at room temperature. After the completion of reaction the mixture was poured into finely crushed ice with stirring and extracted with ethyl acetate (4×25 mL). The combined organic layer was washed with 5% HCl, 5% sodium hydroxide and finally with aqueous NaCl solution. The organic layer was dried over anhydrous magnesium sulphate, filtered and the solvent was removed under reduced pressure to afford the crude products **4a-4f**. The title compounds **4a-f** were purified by silica gel column chromatography (n-hexane: ethyl acetate 4:1). The same procedure was used for the preparation of compounds **6a-d**.

**2-[2-methyl-5-(propan-2-yl)phenoxy]-2-oxoethyl 3-hydroxybenzoate (4a)** solid; reaction time, 24h; yield, 85%; melting point, 77–79°C; R_f_ = 0.56 (n-hexane:ethyl acetate 3:1), FTIR ν_max_ cm^-1^: 3165 (O-H), 3012 (sp2 C-H), 2987 (sp3 C-H), 1721 (C = O ester), 1598 (C = C aromatic), 1147 (C-O, ester); ESI-MS: *m/z* 351 [M + 23] (M + Na)^+^; ^1^H NMR (DMSO-*d*_6_, δ ppm): 7.70 (d, *J* = 5.2 Hz, 1H, H-6), 7.48 (s, 1H, H-2), 7.39 (dd, *J* = 5.2, 7.4 Hz, 1H, H-5), 7.31 (d, *J* = 7.4 Hz, 1H, H-4), 7.11 (d, *J* = 2.6 Hz, 1H, H-3’), 7.01 (d, *J* = 7.4 Hz, 1H, H-4’), 6.92 (s, 1H, H-6’), 5.13 (s, 2H, -CH_2_), 3.03 (sept, 1H, *J* = 7.2 Hz, H-1”), 2.30 (s, 3H, H-3”), 1.38 (s, 1H, -OH), 1.23 (d, *J* = 7.2 Hz, 6H, H-2”); ^13^C NMR (CDCl_3_, δ ppm); 166.3 (C = O ester), 165.8 (C = O, ester), 155.8 (C-3), 146.5 (C-1’), 137.4 (C-2’), 135.9 (C-5’), 132.4 (C-6), 130.8 (C-2), 128.7 (C-1), 126.9 (C-3’), 123.7 (C-4’), 121.6 (C-6’), 119.4 (C-4), 115.8 (C-5), 62.5 (-CH_2_), 29.6 (C-1”), 27.8 (C-3”), 22.8 (C-2”); Anal Calcd For C_19_H_20_O_5_: C, 69.51; H, 6.10; Found C, 69.47; H, 6.14

**2-[2-methyl-5-(propan-2-yl)phenoxy]-2-oxoethyl 4-hydroxybenzoate (4b)** solid; reaction time, 24h; yield, 84%; melting point, 85–87°C; R_f_ = 0.54 (n-hexane:ethyl acetate 2:1), FTIR ν_max_ cm^-1^: 3137 (O-H), 29343 (sp2 C-H), 2876 (sp3 C-H), 1726 (C = O ester), 1598 (C = C aromatic), 1152 (C-O, ester); ESI-MS: *m/z* 351 [M + 23] (M + Na)^+^;^1^H NMR (DMSO-*d*_6_, δ ppm): 8.01 (dd, *J* = 7.4, 2.6 Hz, 2H, H-2, H-6), 7.26 (d, *J* = 2.4 Hz, 1H, H-2’), 7.20 (dd, *J* = 7.6, 2.4 Hz, 1H, H-4’), 7.07 (d, *J* = 7.6, 1H, H-6’), 6.89 (dd, *J* = 7.4, 2.4 Hz, 2H, H-3, H-5), 5.12 (s, 2H, -CH_2_), 4.97 (s, 1H, -OH), 2.86 (sept, *J* = 6.8 Hz, 1H, H-1”), 2.19 (s, 3H, -H-3”),), 1.21 (d, *J* = 6.8 Hz, 6H, H-2”); ^13^C NMR (DMSO-*d*_6_, δ ppm); 168.6 (C = O ester), 166.4 (C = O, ester), 158.5 (C-4), 151.4 (C-1’), 138.1 (C-2’), 137.4 (C-5’), 134.5 (C-3, C-5), 129.3 (C-1), 127.2 (C-3’), 124.2 (C-4’), 122.6 (C-6’), 117.7 (C-2,C-6), 62.3 (-CH_2_), 29.6 (C-1”), 27.2 (C-3”), 23.4 (C-2”); Anal Calcd For C_19_H_20_O_5_: C, 69.51; H, 6.10; Found C, 69.55; H, 6.04

**2-[2-methyl-5-(propan-2-yl)phenoxy]-2-oxoethyl 2,4-dihydroxybenzoate(4c)** solid; reaction time, 24h; yield, 81%; melting point, 106–108°C; R_f_ = 0.49 (n-hexane:ethyl acetate 2:1), FTIR ν_max_ cm^-1^: 3133 (O-H), 2963 (sp2 C-H), 2898 (sp3 C-H), 1723 (C = O ester), 1611 (C = C aromatic), 1165 (C-O, ester); ESI-MS: *m/z* 367 [M + 23] (M + Na)^+^; ^1^H NMR (DMSO-*d*_6_, δ ppm): 7.75 (d, *J* = 8.8 Hz, 1H, H-6), 7.22 (d, *J* = 8.8 Hz, 1H, H-5), 7.10 (d, *J* = 2.4 Hz, 1H, H-6’), 6.91 (s, 1H, H-3), 6.38 (d, *J* = 6.4 Hz, 1H, H-3’), 6.36 (dd, *J* = 6.4, 2.4 Hz, 1H, H-4’), 5.22 (s, 2H, -CH_2_), 4.78 (s, 2H, -OH), 2.90 (sept, *J* = 6.8 Hz, 1H, H-1”), 2.12 (s, 3H, H-3”), 1.18 (d, *J* = 6.8 Hz, 6H, H-2”); ^13^C NMR (DMSO-*d*_6_, δ ppm); 167.3 (C = O ester), 166.5 (C = O ester), 164.6 (C-2), 160.3 (C-4), 149.5 (C-1’), 137.3 (C-2’), 135.3 (C-5’), 133.5 (C-6), 128.2 (C-4’), 126.4 (C-3’), 121.5 (C-6’), 110.8 (C-3), 106.4 (C-5), 101.7 (C-1), 58.8 (-CH_2_), 28.9 (C-1”), 26.4 (C-3”), 22.6 (C-2”); Anal Calcd For C_19_H_20_O_6_: C, 66.28; H, 5.81; Found C, 66.21; H, 5.75

**2-[2-methyl-5-(propan-2-yl)phenoxy]-2-oxoethyl 3,4-dihydroxybenzoate(4d)** solid; reaction time, 24h; yield, 78%; melting point, 162–164°C; R_f_ = 0.46 (n-hexane:ethyl acetate 2:1), FTIR ν_max_ cm^-1^: 3166 (O-H), 2983 (sp2 C-H), 2856 (sp3 C-H), 1729 (C = O ester), 1596 (C = C aromatic), 1137 (C-O, ester); ESI-MS: *m/z* 367 [M + 23] (M + Na)^+^; ^1^H NMR (DMSO-*d*_6_, δ ppm): 7.76 (d, *J* = 2.1 Hz, 1H, H-2), 7.46 (dd, *J* = 7.3, 2.1 Hz, 1H, H-6), 7.30 (d, *J* = 7.3 Hz, 1H, H-5), 7.11 (d, *J* = 1.2 Hz, 1H, H-5’), 6.98 (d, *J* = 7.4 Hz, 1H, H-3’), 6.89 (dd, *J* = 7.4, 1.2 Hz, 1H, H-4’), 5.17 (s, 2H, -CH_2_), 4.47 (s, 2H, -OH), 2.76 (sept, *J* = 6.4 Hz, 1H, H-1”), 2.21 (s, 3H, H-3”), 1.16 (d, *J* = 6.4 Hz, 6H, H-2”); ^13^C NMR (DMSO-*d*_6_, δ ppm); 168.2 (C = O ester), 166.3 (C = O ester), 155.8 (C-3), 148.3 (C-4), 145.1 (C-1’), 139.1 (C-2’), 137.3 (C-5’), 129.5 (C-6), 127.3 (C-4’), 124.5 (C-3’), 121.4 (C-6’), 120.3 (C-2), 117.3 (C-5), 113.4 (C-1), 62.8 (-CH_2_), 28.2 (C-1”), 25.1 (C-3”), 21.5 (C-2”); Anal Calcd For C_19_H_20_O_6_: C, 66.28; H, 5.81; Found C, 66.35; H, 5.87

**2-[2-methyl-5-(propan-2-yl)phenoxy]-2-oxoethyl 3,5-dihydroxybenzoate(4e)** solid; reaction time, 24h; yield, 86%; melting point, 116–118°C; R_f_ = 0.48 (n-hexane:ethyl acetate 2:1), FTIR ν_max_ cm^-1^: 3145 (O-H), 2956 (sp2 C-H), 2843 (sp3 C-H), 1719 (C = O ester), 1601 (C = C aromatic), 1148 (C-O, ester); ESI-MS: *m/z* 367 [M + 23] (M + Na)^+^; ^1^H NMR (DMSO-*d*_6_, δ ppm): 7.22 (d, *J* = 8.0 Hz, 1H, H-3’), 7.11 (d, *J* = 8.0 Hz, 1H, H-4’), 7.01 (d, *J* = 2.2 Hz, 2H, H-3,H-5), 6.90 (d, *J* = 2.2 Hz, 1H, H-4), 6.52 (s, 1H, H-6’), 5.18 (s, 2H, -CH_2_), 4.87 (s, 2H, -OH), 2.93 (sept, *J* = 7.3 Hz, 1H, H-1”), 2.27 (s, 3H, H-3”), 1.06 (d, *J* = 7.3 Hz, 6H, H-2”); ^13^C NMR (DMSO-*d*_6_, δ ppm); 167.5 (C = O ester), 165.6 (C = O ester), 160.2 (C-3, C-5), 158.5 (C-1’), 148.4 (C-2’), 137.4 (C-5’), 131.5 (C-2, C-6), 128.3 (C-4’), 124.2 (C-3’), 121.4 (C-6’), 110.4 (C-4), 106.3 (C-1), 60.4 (-CH_2_), 28.3 (C-1”), 27.5 (C-3”), 23.7 (C-2”); Anal Calcd For C_19_H_20_O_6_: C, 66.28; H, 5.81; Found C, 66.19; H, 5.88.

**2-[2-methyl-5-(propan-2-yl)phenoxy]-2-oxoethyl 3,4,5-trihydroxybenzoate(4f)** solid; reaction time, 24h; yield, 87%; melting point, 136–138°C; R_f_ = 0.52 (n-hexane:ethyl acetate 2:1), FTIR ν_max_ cm^-1^: 3187 (O-H), 2963 (sp2 C-H), 2885 (sp3 C-H), 1721 (C = O ester), 1598(C = C aliphatic) 1612 (C = C aromatic), 1152 (C-O, ester); ESI-MS: *m/z* 383 [M + 23] (M + Na)^+^; ^1^H NMR (DMSO-*d*_6_, δ ppm): 7.21 (d, *J* = 7.6 Hz, 1H, H-3’), 7.01 (d, *J* = 7.6 Hz, 1H, H-4’), 6.54 (s, 2H, H-2, H-6), 6.43 (s, 1H, H-6’), 5.12 (s, 2H, -CH_2_), 4.47 (s, 2H, -OH), 2.86 (sept, *J* = 6.5 Hz, 1H, H-1”), 2.27 (s, 3H, H-3”), 1.16 (d, *J* = 6.5 Hz, 6H, H-2”); ^13^C NMR (DMSO-*d*_6_, δ ppm); 166.2 (C = O ester), 165.4 (C = O ester), 160.5 (C-3,C-4, C-5), 158.5 (C-1’), 151.4 (C-2’), 138.3 (C-5’), 134.2 (C-2, C-6), 128.3 (C-4’), 126.4 (C-3’), 121.6 (C-6’), 110.5 (C-4), 103.5 (C-1), 60.3 (-CH_2_), 29.5(C-1”), 26.8 (C-3”), 22.3 (C-2”); Anal Calcd For C_19_H_20_O_6_: C, 66.28; H, 5.81; Found C, 66.19; H, 5.89

**2-[2-methyl-5-(propan-2-yl)phenoxy]-2-oxoethyl (2E)-3-phenylprop-2-enoate(6a)** solid; reaction time, 24h; yield, 87%; melting point, 94–96°C; R_f_ = 0.54 (n-hexane:ethyl acetate 2:1), FTIR ν_max_ cm^-1^: 2987 (sp2 C-H), 2865 (sp3 C-H), 1729 (C = O ester), 1598 (C = C aromatic), 1151 (C-O, ester); ESI-MS: *m/z* 361 [M + 23] (M + Na)^+^; ^1^H NMR (DMSO-*d*_6_, δ ppm): 7.84 (d, *J* = 16.0 Hz, 1H, H-2), 7.51 (dd, *J* = 7.8, 2.2 Hz, 2H, H-2’, H-6’), 7.36–7.42 (m, 3H, H-3’, H-4’, H-5’), 7.18 (d, *J* = 6.8 Hz, 1H, H-3”), 7.06 (d, *J* = 6.8 Hz, 1H, H-4”), 6.81 (s, 1H, H-6”), 6.55 (d, *J* = 16.0 Hz, 1H, H-1), 5.07 (s, 2H, -CH_2_), 2.98 (sept, *J* = 7.2 Hz, 1H, H-1”‘), 2.31 (s, 3H, H-3”‘), 1.13 (d, *J* = 7.2 Hz, 6H, H-2”‘); ^13^C NMR (DMSO-*d*_6_, δ ppm); 168.2 (C = O ester), 165.4 (C = O, ester), 148.4 (C-1”), 144.3 (C-2), 135.7 (C-2”), 133.7 (C-5”), 131.6 (C-2’,C-6’), 127.5 (C-3’, C-5’), 126.3 (C-4’), 125.3 (C-1’), 123.8 (C-3”), 120.5 (C-4”), 117.2 (C-6”), 62.1 (-CH_2_), 28.5 (C-1”‘), 22.5 (C-3”‘), 19.8 (C-2”‘); Anal Calcd For C_21_H_22_O_4_: C, 74.56; H, 6.51; Found C, 74.47; H, 6.62.

**2-[2-methyl-5-(propan-2-yl)phenoxy]-2-oxoethyl (2E)-3-(4-hydroxyphenyl)prop-2-enoate(6b)** solid; reaction time, 24h; yield, 80%; melting point, 117–119°C; R_f_ 0.46 (n-hexane:ethyl acetate 2:1), FTIR ν_max_ cm^-1^: 3178 (-OH), 2968 (sp2 C-H), 2889 (sp3 C-H), 1721 (C = O), 1601 (C = C aromatic), 1139 (C-O, ester); ESI-MS: *m/z* 377 [M + 23] (M + Na)^+^; ^1^H NMR (DMSO-*d*_6_, δ ppm):7.68 (d, *J* = 16.0 Hz, 1H, H-2), 7.51 (dd, *J* = 7.4, 2.2 Hz, 2H, H-2’, H-6’), 7.42 (dd, *J* = 7.4, 2.2 Hz, 2H, H-3’, 5’), 7.22 (d, *J* = 7.6 Hz, 1H, H-3”), 7.13 (d, *J* = 7.6 Hz, 1H, H-4”), 6.94 (s, 1H, H-6”), 6.51 (d, *J* = 16.0 Hz, 1H, H-1), 5.07 (s, 2H, -CH_2_), 3.01 (sept, *J* = 7.1 Hz, 1H, H-1”‘), 2.29 (s, 3H, H-3”‘), 1.18 (d, *J* = 7.1 Hz, 6H, H-2”‘); ^13^C NMR (DMSO-*d*_6_, δ ppm); 168.4 (C = O ester), 165.8 (C = O, ester), 159.2 (C-1”), 145.6 (C-2), 143.2 (C-2”), 137.3 (C-5”), 131.5 (C-3’, C-5’), 128.4 (C-4’), 125.3 (C-1’), 122.5 (C-3”), 120.6 (C-4”), 116.5 (C-2’,C-6’), 113.7 (C-6”), 58.6 (-CH_2_), 29.1 (C-1”‘), 27.1 (C-3”‘), 23.0 (C-2”‘); Anal Calcd For C_21_H_22_O_5_: C, 71.19; H, 6.21; Found C, 71.26; H, 6.10.

**2-[2-methyl-5-(propan-2-yl)phenoxy]-2-oxoethyl (2E)-3-(2,4-dihydroxyphenyl)prop-2-enoate(6c)** solid; reaction time, 24h; yield, 76%; melting point, 129–1319°C; R_f_ 0.45 (n-hexane:ethyl acetate 2:1), FTIR ν_max_ cm^-1^: 3189 (-OH), 2987 (sp2 C-H), 2867 (sp3 C-H), 1729 (C = O), 1611 (C = C aromatic), 1151 (C-O, ester); ESI-MS: *m/z* 393 [M + 23] (M + Na)^+^; ^1^H NMR (DMSO-*d*_6_, δ ppm): 7.63 (d, *J* = 16.0 Hz, 1H, H-2), 7.53 (d, *J* = 7.6, Hz, 1H, H-6’), 7.46 (d, *J* = 7.6, Hz, 1H, H-5’), 7.40 (s, 1H, H-3’), 7.28 (d, *J* = 6.6 Hz, 1H, H-3”), 7.02 (d, *J* = 6.6 Hz, 1H, H-4”), 6.94 (s, 1H, H-6”), 6.56 (d, *J* = 16.0 Hz, 1H, H-1), 5.04 (s, 2H, -CH_2_), 3.08 (sept, *J* = 7.2 Hz, 1H, H-1”‘), 2.38 (s, 3H, H-3”‘), 1.28 (d, *J* = 7.2 Hz, 6H, H-2”‘); ^13^C NMR (DMSO-*d*_6_, δ ppm); 167.8 (C = O ester), 166.5 (C = O, ester), 158.8 (C-1”), 149.2 (C-2), 143.3 (C-2”), 135.2 (C-5”), 132.2 (C-3’), 128.3 (C-5’), 128.5 (C-4’), 127.2 (C-1’), 126.8 (C-3”), 124.3 (C-4”), 119.5 (C-2’), 117.4 (C-6’), 110.4 (C-6”), 55.4 (-CH_2_), 29.5 (C-1”‘), 28.6 (C-3”‘), 24.2 (C-2”‘); Anal Calcd For C_21_H_22_O_5_: C, 71.19; H, 6.21; Found C, 71.27; H, 6.14.

**2-[2-methyl-5-(propan-2-yl)phenoxy]-2-oxoethyl (2E)-3-(4-chlorophenyl)prop-2-enoate(6d)** solid; reaction time, 24h; yield, 84%; melting point, 83–85°C; R_f_ 0.58 (n-hexane:ethyl acetate 2:1), FTIR ν_max_ cm^-1^:2989 (sp2 C-H), 2891 (sp3 C-H), 1734 (C = O ester), 1615 (C = C aromatic), 1149 (C-O, ester); ESI-MS: *m/z* 395 [M + 23] (M + Na)^+^; ^1^H NMR (DMSO-*d*_6_, δ ppm): 7.72 (d, *J* = 16.0 Hz, 1H, H-2), 7.48 (d, *J* = 7.4 Hz, 2H, H-2’, 6’), 7.31 (d, *J* = 8.0 Hz, 1H, H-3”), 7.12 (d, *J* = 8.0 Hz, 1H, H-4”), 6.93 (s, 1H, H-6”), 6.87 (d, *J* = 7.4 Hz, 2H, H-3’, H-5’), 6.47 (d, *J* = 16.0 Hz, 1H, H-1), 5.04 (s, 2H, -CH_2_), 3.01 (sept, *J* = 7.4 Hz, 1H, H-1”‘), 2.56 (s, 3H, H-3”‘), 1.21 (d, *J* = 7.4 Hz, 6H, H-2”‘); ^13^C NMR (CDCl_3_, δ ppm); 168.4 (C = O ester), 166.2 (C = O, ester), 151.3 (C-1”), 146.5 (C-2), 138.3 (C-2”), 136.3 (C-5”), 133.5 (C-2’,C-6’), 130.3 (C-3’, C-5’), 129.5 (C-4’), 128.1 (C-1’), 125.6 (C-3”), 123.9 (C-4”), 119.2 (C-6”), 60.5 (-CH_2_), 28.5 (C-1”‘), 26.4 (C-3”‘), 23.2 (C-2”‘); Anal Calcd For C_21_H_21_O_4_Cl: C, 67.65; H, 5.64; Found C, 67.56; H, 5.75.

### Anti-tyrosinase activity

The mushroom tyrosinase (EC 1.14.18.1) (Sigma Chemical Co.) was used for in vitro bioevaluation as described previously with some modifications [40–41]. Briefly, 140μL of phosphate buffer (20mM, pH 6.8), 20μL of mushroom tyrosinase (30U/mL) and 20μL of the inhibitor solution were placed in the wells of a 96-well micro plate. After pre-incubation for 10 min at room temperature, 20μL of L-DOPA (3,4-dihydroxyphenylalanine) (0.85mM) was added and the plate was further incubated at 25°C for 20min. Subsequently the absorbance of dopachrome was measured at 492nm using a micro plate reader (OPTI_Max_, Tunable). Kojic acid was used as a reference inhibitor and for negative tyrosinase inhibitor phosphate buffer was used instead of the inhibitor solution. The extent of inhibition by the synthesized carvacrol derivatives **4a-f** and **6a-d** was expressed as the percentage of concentration necessary to achieve 50% inhibition (IC_50_). Each concentration was analyzed in three independent experiments run in triplicate. The IC_50_ values were determined by the data analysis and graphing software Origin 8.6, 64-bit.

#### Kinetic analysis of tyrosinase inhibition activity

The carvacrol derivatives **4c, 6b** and **6c** were selected to determine their kinetic mechanism of enzyme inhibition.A series of experiments were performed to determine the inhibition kinetics by following the already reported method [42–43]. Inhibitor **6b** with concentrations 0.0, 7.0, 28 μM, in case of Lineweaver-Burk plot and with concentrations0.0, 1.75, 7.0, 14.0, 28 μM in case of Dixon plot was used respectively. Compound **6c** with concentrations0.0, 0.01, 0.02, 0.04μMand **4c** with concentrations 0.0, 1.68, 3.37, 6.75, 13.5 μM,respectively were usedin case of Lineweaver-Burk plot as well as in Dixon plot. Substrate L-DOPA concentration was among 0.0625 to 2 mM in all kinetic study. Pre-incubation and measurement time was the same as discussed in mushroom tyrosinase inhibition assay protocol. Formation of DOPAchrome was continuously monitored at 475nm for 5min at a 30s interval in the microplate reader after addition of enzyme. The inhibition type on the enzyme was assayed by Lineweaver-Burk plots of inverse of velocities 1/*V* versus inverse of substrate concentration 1/[S] mM^-1^, and the inhibition constant *Ki* was determined by Dixon plot of 1/*V* versus inhibitor concentrations.

The effect of concentrations of compounds **4c, 6b** and **6c** on the activity of mushroom tyrosinase for the catalysis of DOPA at 37°C was also studied. Assay conditions were same as described in assay protocolexceptthat the final concentration of enzyme was varied (2 to 10 μg/mL). Concentrations of compounds **6b**(0.0, 1.75, 3.5, 7.0, 14.0, 28, 56 μM) **6c**(0.001, 0.005, 0.01, 0.02, 0.04 μM)and **4c**(0.0, 1.7, 3.4, 6.7, 13.5, 27, 54 μM)were used respectively, for the determination of reversible as well as irreversible binding of inhibitors at enzyme.

### Molecular docking studies

#### Retrieval of target structure

The crystal structure of mushroom tyrosinase (PDBID: 2Y9X) was retrieved from Protein Data Bank (PDB) (http://www.rcsb.org). The energy minimizations target protein was carried out by employing conjugate gradient algorithm and Amber force field in UCSF Chimera 1.10.1 [44]. The stereo-chemical properties, Ramachandran graph and values [45] of mushroom tyrosinase structure were assessed by Molprobity server[46], while the hydrophobicity graph was generated by Discovery Studio 4.1 Client [47]. The protein architecture and statistical percentage values of helices, beta-sheets, coils and turn were accessed by using online tool VADAR 1.8 [48].

#### Candidate structures

The synthesized candidate molecules **4a-f** and **6a-d** were sketched in drawing ACD/ChemSketch tool. The designed ligand molecules were further visualized and minimized by UCSF Chimera 1.10.1. Multiple online drug assessment computational tools like Molinspiration(http://www.molinspiration.com/) and Molsoft (http://www.molsoft.com/) were used to predict the drug-likeness and biological properties of these designed candidate molecules. The Osiris Property Explorer was used to evaluate their possible tumorigenic or mutagenic risks and also to calculate the drug-likeness values. Lipinski’s rule of five was analyzed using Molsoft and Molinspiraion tools. Furthermore, their predicted Absorption, Distribution, Metabolism, Excretion and Toxicity (ADMET) properties were evaluated by pkCSM online tool [49]. Similarly, different molecular properties like Molar Refractivity, Density, Surface tension and Polarizability were accessed by ChemSketch.

#### Molecular docking

Molecular docking of all the synthesized ligand molecules **4a-f** and **6a-d** against mushroom tyrosinase were carried out using diverse AutoDock 4.2 tool according to the specified instructions [50]. In brief, for receptor protein the polar hydrogen atoms and Kollman charges were apportioned and for ligand, Gasteiger partial charges were designated and non-polar hydrogen atoms were merged. All the torsion angles for all the ligands were set free to rotate through docking experiments. The docking experiments were carried out by considering receptor as a rigid while ligands as flexible molecules. A grid map of 80 Åx80 Åx80 Å was set on the whole protein structure to generate the grid map. The number of runs for each docking experiment was set to 100. The Lamarckian genetic algorithm (LGA) and empirical free energy function were applied by taking docking parameters default. The docked complexes were further evaluated on lowest binding energy (Kcal/mol) values and hydrogen bond analysis using Discovery Studio (4.1) and UCSF Chimera 1.10.1.

## Supporting information

S1 Fig Synthesis of carvacrol derivatives (4a-f). (TIF)

S2 Fig Synthesis of carvacrol derivatives (6a-d). (TIF)
