# Supplementary figures and images for: Carvacrol derivatives as mushroom tyrosinase inhibitors; synthesis, kinetics mechanism and molecular docking studies
Source: PLoS One. 2017 May 23;12(5):e0178069. doi: 10.1371/journal.pone.0178069 (PMC5441849; doi:10.1371/journal.pone.0178069)

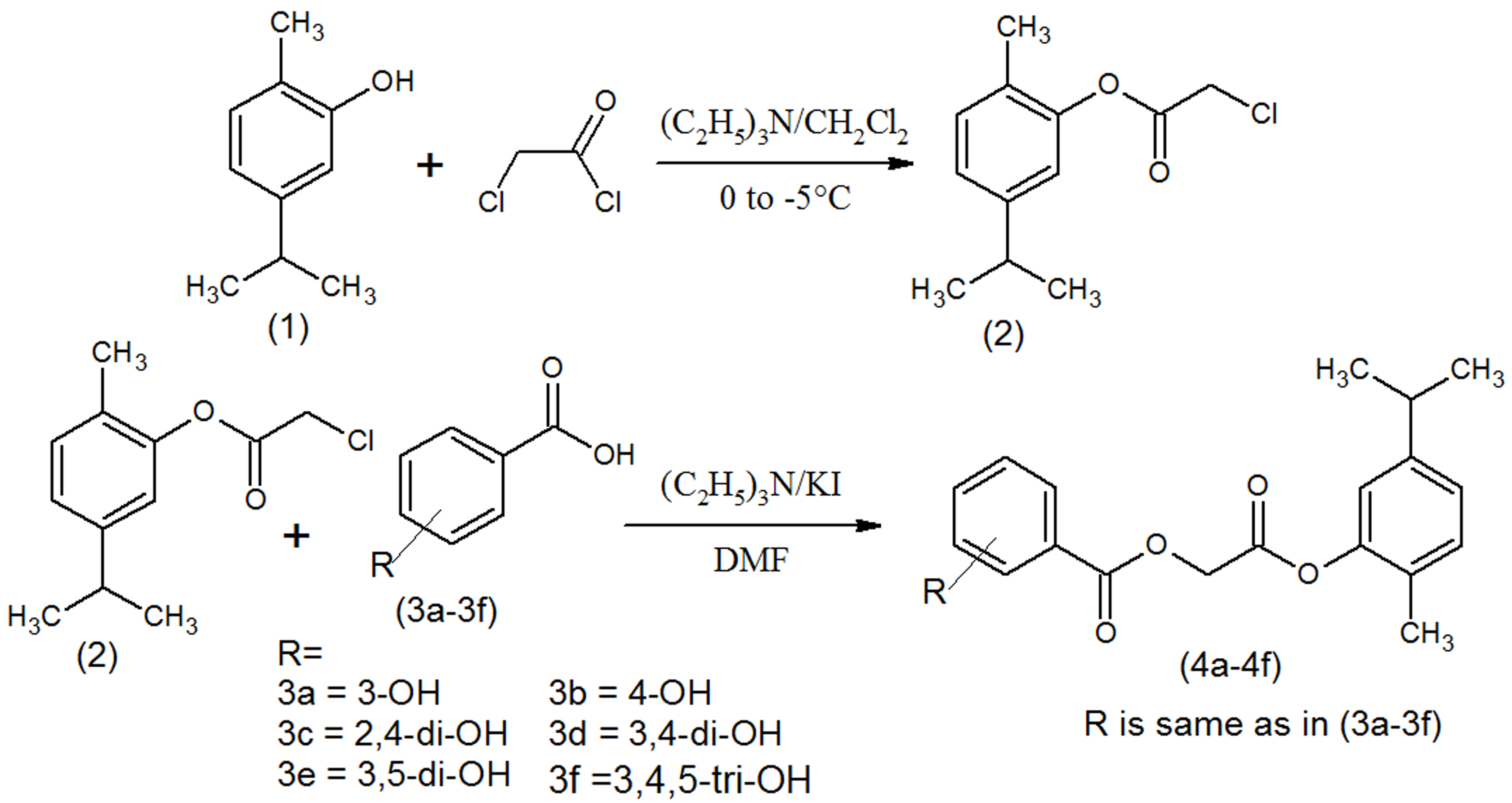

Supplement: S1 Fig — (TIF) [file pone.0178069.s001.tif]

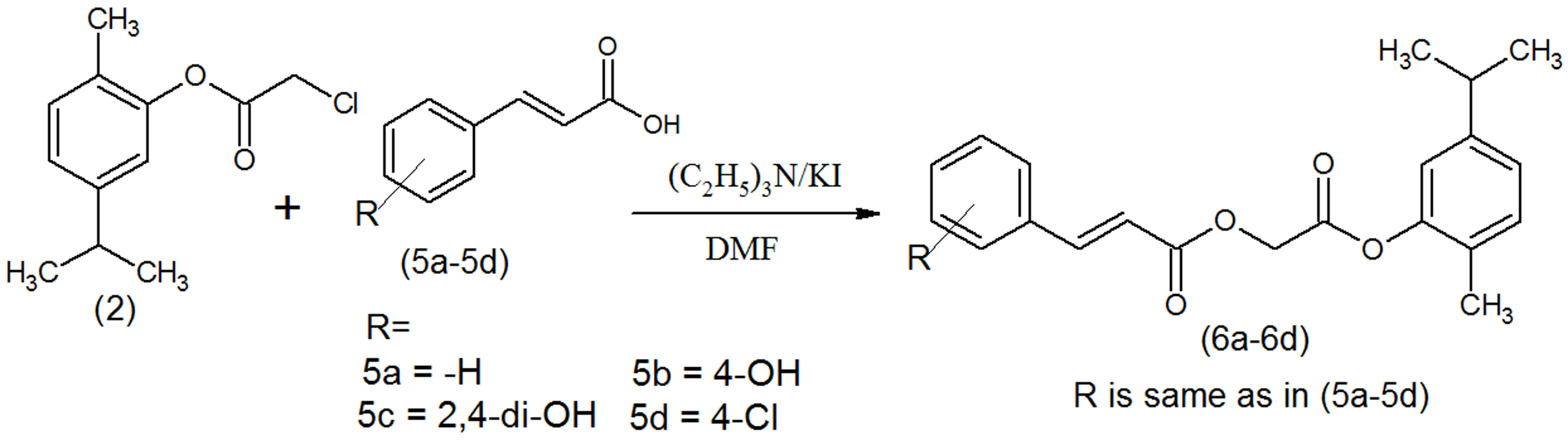

Supplement: S2 Fig — (TIF) [file pone.0178069.s002.tif]
